# Interleukin family in vascular calcification: molecular mechanisms and therapeutic perspectives

**DOI:** 10.3389/fcvm.2025.1619018

**Published:** 2025-09-01

**Authors:** Yikun Zhao, Heng Li, Yuanyuan Guo

**Affiliations:** Vascular Surgery Department, Fuwai Yunnan Cardiovascular Hospital, Kunming Medical University, Kunming, China

**Keywords:** interleukin family, vascular calcification, osteogenic differentiation, inflammation, therapeutic targets

## Abstract

Vascular calcification (VC), characterized by pathological calcium deposition in arterial walls, is a major contributor to cardiovascular morbidity in chronic inflammatory diseases such as atherosclerosis, chronic kidney disease (CKD), and diabetes. Emerging evidence underscores the pivotal role of interleukin (IL) family cytokines in modulating VC through dual pro- and anti-calcific mechanisms. Pro-inflammatory IL members, including IL-1β, IL-6, IL-17A, and IL-29, drive osteogenic transdifferentiation of vascular smooth muscle cells (VSMCs) by activating pathways such as NF-κB, STAT3, NLRP3 inflammasomes, and Wnt/β-catenin. These pathways upregulate osteogenic markers (e.g., Runx2, BMP-2) and promote oxidative stress, matrix remodeling, and pyroptosis. Conversely, anti-inflammatory cytokines like IL-10 counteract calcification by suppressing inflammatory signaling, enhancing autophagy, and restoring mineral homeostasis. This review highlights the dynamic interplay between IL cytokines, metabolic dysregulation, and epigenetic modifications in VC pathogenesis. It advocates for multi-target approaches, such as combining TYK2/STAT3 inhibition with metabolic reprogramming, to disrupt pathological crosstalk. Future research must address spatiotemporal heterogeneity in IL signaling and optimize therapeutic specificity to translate mechanistic insights into clinical applications. Harnessing the IL family's dual roles offers transformative potential for mitigating VC while preserving immune integrity.

## Introduction

Vascular calcification (VC), characterized by pathological calcium-phosphate deposition in arterial walls, is a hallmark of cardiovascular morbidity in chronic inflammatory diseases such as atherosclerosis, chronic kidney disease (CKD), and diabetes (1, 2). VC manifests as intimal calcification within atherosclerotic plaques or medial calcification (Mönckeberg's sclerosis), both independently predicting adverse outcomes like myocardial infarction and peripheral artery disease (3, 4). Intimal calcification destabilizes plaques, increasing rupture risk, while medial calcification reduces vascular compliance, exacerbating hypertension and heart failure (5). Patients with CKD exhibit accelerated VC progression due to phosphate metabolism imbalance and chronic inflammation. Among predialysis and late-stage CKD patients, the incidence of coronary artery calcification reaches 64%–77% (6). VC is not merely a passive degenerative process but an active cellular phenomenon involving osteochondrogenic transdifferentiation of vascular smooth muscle cells (VSMCs) and metabolic-inflammatory crosstalk (7). Emerging evidence underscores VC as a dynamic interplay between mineral dysregulation, oxidative stress, and immune activation, positioning it as a critical therapeutic target in cardiovascular pathology (2, 8).

Chronic inflammation is central to VC pathogenesis, with pro-inflammatory cytokines of the interleukin (IL) family orchestrating osteogenic progression of VSMCs (9). IL-1β and IL-6 synergistically activate pathways such as NF-κB and STAT3, upregulating osteogenic markers (e.g., Runx2, BMP-2) and promoting matrix remodeling (9, 10). IL-17A amplifies oxidative stress via Wnt/β-catenin signaling, while IL-18 induces pyroptosis through NLRP3 inflammasome activation, releasing calcification-primed matrix vesicles (11, 12). Conversely, anti-inflammatory cytokines like IL-37 counteract calcification by suppressing NF-κB and enhancing autophagy (13). The IL family's dual roles highlight its regulatory complexity: pro-calcific members drive VSMC senescence and mineralization, while protective cytokines restore mineral homeostasis (14). Clinical studies in CKD patients have shown that elevated IL-6 levels are associated with arterial calcification. Separately, preclinical studies in urotensin-deficient mice indicate that increased IL-1β contributes to this pathology. These findings highlight the prognostic value of both cytokines (15, 16).

This review systematically examines the IL family's dual mechanisms in VC, emphasizing translational potential. We explore how IL-1β, IL-6, and IL-17A promote osteogenic transformation, while IL-10 and IL-37 mitigate calcification via metabolic reprogramming. Emerging strategies, such as IL-6 trans-signaling inhibition (sgp130Fc) and NLRP3 targeting, demonstrate efficacy in preclinical models but face challenges in clinical specificity (15, 17). By integrating molecular insights with clinical evidence, we aim to identify precision therapeutic avenues for modulating IL-driven vascular pathology, addressing spatiotemporal heterogeneity in cytokine signaling and optimizing immune-metabolic balance (4, 18).

**Figure 1:**
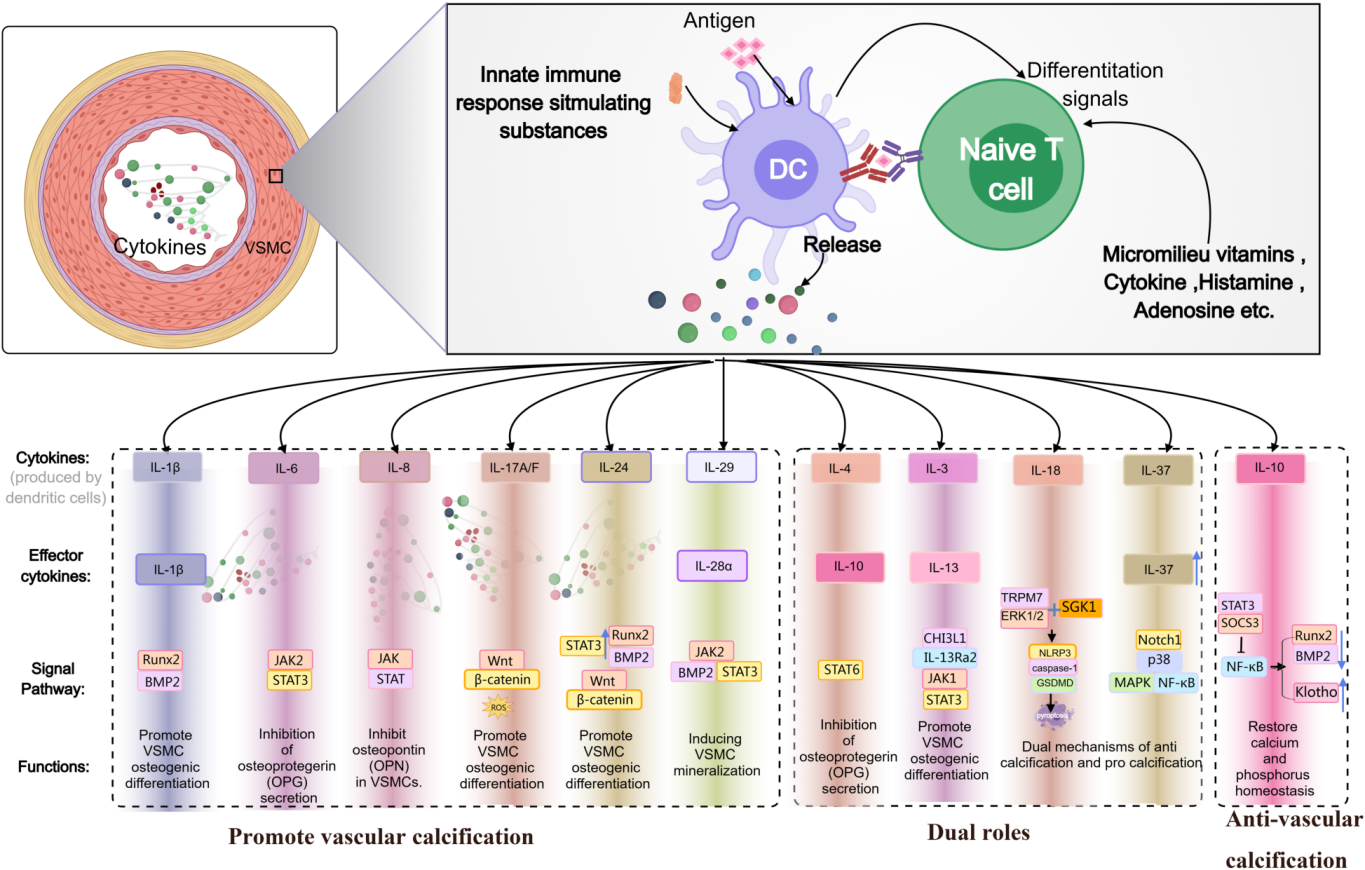
Schematic diagram illustrating the dual roles of IL family members in promoting or inhibiting VC.

This Figure 1 illustrates how dendritic cells (DCs), upon interacting with naïve T cells, release multiple cytokines (e.g., IL-1β, IL-6, IL-8, IL-17A/F, IL-24, IL-29, IL-4, IL-3, IL-18, IL-37, IL-10), which regulate the osteogenic differentiation and calcification of VSMCs via signaling pathways such as BMP2/Runx2, JAK/STAT, and Wnt/β-catenin. Certain cytokines can simultaneously exhibit both pro- and anti-calcific properties, ultimately influencing the progression of VC and calcium-phosphorus homeostasis.

## Molecular mechanisms of vascular calcification

### Osteogenic transdifferentiation of VSMCs

The osteogenic transdifferentiation of VSMCs represents a pivotal pathological event in VC, driven by transcriptional reprogramming, metabolic remodeling, and inflammatory crosstalk. Central to this process is the activation of the master transcriptional regulator Runx2, which orchestrates the expression of osteogenic markers such as bone morphogenetic protein-2 (BMP-2) and alkaline phosphatase (ALP) (19). The synergistic interaction between BMP-2/SMAD and Wnt/β-catenin pathways amplifies calcification: BMP-2 induces SMAD1/5 phosphorylation to facilitate β-catenin nuclear translocation, while Wnt signaling enhances Runx2 transcription via KLF4-PFKFB3-mediated glycolytic reprogramming, providing bioenergetic support for phenotypic switching (20). Recent studies highlight the regulatory role of Cdon, a Wnt antagonist, which attenuates calcification by disrupting β-catenin-Runx2 binding through its Ig2 domain (21). Epigenetic modulation further refines this process, as SIRT6 deacetylates Runx2 to promote its nuclear export and proteasomal degradation, thereby suppressing osteogenic differentiation (19). Pro-inflammatory cytokines, notably IL-29 and IL-6, exacerbate calcification via JAK2/STAT3 signaling, which upregulates BMP-2 expression and enhances Runx2 transcriptional activity through STAT3-Tyr705 phosphorylation (22, 23). Metabolic perturbations, such as O-GlcNAcylation mediated by O-GlcNAc transferase (OGT), stabilize β-catenin to potentiate Wnt signaling, accelerating VSMC mineralization (24). These findings underscore a multidimensional regulatory network, identifying actionable targets—including Cdon-Ig2, SIRT6 activators, and OGT inhibitors—for therapeutic intervention in VC.

The progression of VC is governed by synergistic interactions between metabolic derangements and inflammatory signaling. Hyperphosphatemia activates monocytes via the phosphate transporter PiT-1, triggering TNF-α and IL-6 release to establish a pro-inflammatory microenvironment that accelerates VSMC osteogenic differentiation (25). Phosphate overload synergizes with IL-6 to induce senescence-associated calcification through the IL-6/sIL-6R/STAT3/p53 axis, a process partially reversible by resveratrol (26). Emerging evidence highlights the role of leukemia inhibitory factor (LIF) in amplifying phosphate-driven calcification via the TYK2/STAT3 pathway, with TYK2 inhibition by deucravacitinib—a clinically approved immunosuppressant—significantly attenuating VC in CKD models (27). Furthermore, caspase-1 inhibitor VX-765 mitigates hyperphosphatemia-induced VSMC calcification by suppressing STAT3 phosphorylation and pyroptosis-related NLRP3/GSDMD activation (11). These findings underscore the therapeutic potential of targeting metabolic-inflammatory hubs, particularly TYK2 and STAT3 signaling nodes, to disrupt pathological crosstalk in VC.

Emerging evidence highlights the complexity of VC as a process governed by intersecting epigenetic, metabolic, and inflammatory axes. Recent studies identify SIRT6 and SIRT7 as critical epigenetic regulators that mitigate osteogenic transdifferentiation by deacetylating Runx2 and suppressing oxidative stress-induced senescence, respectively (19, 28). Metabolic dysregulation is further modulated by mitochondrial phosphate carrier (PiC), which drives calcification via ERK1/2-mTOR-dependent superoxide generation, while inhibition of PiC with butyl malonate attenuates VC in CKD model (29). The discovery of ARSE as a promoter of VSMC calcification through Wnt/β-catenin signaling underscores the role of genetic variants in VC pathogenesis, with ARSE knockdown reducing aortic mineralization (30). Therapeutic innovation is exemplified by 4-octyl itaconate (4-OI), which activates HMOX-1 to counteract oxidative stress and inflammation in calcified VSMCs (31), and Moscatilin, a natural compound targeting IL13RA2/STAT3-WNT3/β-catenin crosstalk to inhibit osteogenic differentiation (32)^.^ Additionally, calprotectin inhibition via paquinimod disrupts RAGE/TLR4 signaling, offering a translational strategy for CKD-associated VC (33). These findings collectively map a multidimensional network encompassing mitochondrial dynamics, epigenetic reprogramming, and inflammatory cascades, positioning targeted inhibition of PiC, ARSE, and calprotectin as promising avenues for clinical intervention.

## Role of inflammation and oxidative stress

### Pro-inflammatory mechanisms and targeting strategies in vascular calcification

#### IL-1β

IL-1β, a pivotal pro-inflammatory cytokine, drives VC through NLRP3 inflammasome-pyroptosis axis activation and transcriptional reprogramming of VSMCs. Under hyperphosphatemic conditions, IL-1β activates NF-κB signaling to upregulate Runx2 and BMP2 expression, promoting osteogenic transdifferentiation of VSMCs (34). Clinical studies demonstrate elevated serum IL-1β levels in CKD patients correlate with coronary calcification scores, while NLRP3 inhibition with MCC950 suppresses IL-1β secretion and attenuates calcification (35). Mechanistically, IL-1β induces gasdermin D-mediated pyroptosis, releasing calcification-primed matrix vesicles (MVs) as nucleation sites—a process reversible by caspase-1 inhibitor VX-765 (11). Recent advances reveal a feedforward loop between IL-1β and transcription factor TCF21: TCF21 enhances IL-1β production via ERK1/2-β-catenin signaling, while IL-1β amplifies osteogenic gene transcription through STAT3 activation (9). Therapeutically, the natural compound ligustrazine mitigates coronary calcification by inhibiting caspase-3/GSDME-dependent pyroptosis and IL-1β release in murine models (36). Additionally, Elabela, an endogenous peptide, suppresses IL-1β-driven inflammation via PPAR-γ/FDX1 signaling, offering a novel regulator*y* axis for VC intervention (37). These findings not only delineate IL-1β's multifaceted role in VC but also validate NLRP3 inhibitors (e.g., MCC950), pyroptosis blockers (e.g., VX-765), and natural compounds as promising translational strategies targeting IL-1β signaling.

#### IL-6

IL-6 emerges as a pivotal mediator in VC, driving osteogenic transdifferentiation of VSMCs via the JAK2/STAT3 signaling axis. Clinical studies demonstrate that elevated serum IL-6 levels in CKD patients correlate with coronary artery calcification (CAC) progression and cardiovascular mortality, with high IL-6 tertiles exhibiting a 2.2-fold increased risk of death (38). Mechanistically, IL-6 induces STAT3-Tyr705 phosphorylation, promoting Runx2 nuclear translocation and BMP2 upregulation while suppressing osteoprotegerin (OPG) secretion, thereby disrupting the mineralization balance (22). Hyperphosphatemia exacerbates this process by activating PiT-1 receptors on monocytes, triggering IL-6 release and establishing a self-reinforcing inflammatory-calcific loop (25). Recent advances reveal a feedforward mechanism involving transcription factor TCF21, which enhances IL-6 expression via ERK1/2-β-catenin signaling, while IL-6 reciprocally amplifies STAT3 activation to potentiate osteogenic gene transcription (9). Therapeutic interventions targeting this axis show promise: JAK inhibitors like tofacitinib attenuate aortic calcification by blocking IL-6R/gp130 signaling (39), while the natural compound Ptd-1 suppresses IL-6/STAT3 crosstalk and ERK/β-catenin interactions to reduce VSMC mineralization (40). Additionally, the SGLT2 inhibitor empagliflozin mitigates IL-6-driven inflammation through AMPK/Nrf2 pathway activation, highlighting its potential in CKD-associated VC (41). These findings underscore IL-6 as a multidimensional therapeutic target, with combinatorial strategies addressing both inflammatory and metabolic pathways offering novel avenues for clinical translation.

IL-6 inhibitors, exemplified by tocilizumab, demonstrate significant efficacy in managing various inflammatory conditions but carry notable risks. In polyarticular or systemic juvenile idiopathic arthritis (pJIA/sJIA), long-term subcutaneous tocilizumab maintained disease control, achieving inactive disease in up to 92% of sJIA patients; however, serious adverse events, including infections, occurred in 13.6% of pJIA and 13.2% of sJIA patients (42). For refractory Takayasu arteritis, tocilizumab provided a substantial steroid-sparing effect, with 46.4% of patients reducing glucocorticoid doses to less than half their pre-study relapse dose, alongside improved quality-of-life metrics and no new safety signals during extended treatment (43). In giant cell arteritis, intravenous tocilizumab (6–7 mg/kg) sustained remission without flares, though 16.7% of patients experienced serious adverse events (44). These findings underscore its potent anti-inflammatory effects balanced against infection-related risks.

#### IL-8

IL-8, a potent chemoattractant cytokine, accelerates VC by orchestrating neutrophil infiltration and matrix metalloproteinase-9 (MMP-9)-mediated degradation of calcification inhibitors. In CKD, elevated serum IL-8 levels correlate with VC severity, driven by hyperphosphatemia-induced endothelial cell (EC) secretion of IL-8, which suppresses osteopontin (OPN) expression in VSMCs and compromises intrinsic anti-calcific defenses (45). Mechanistically, phosphate overload activates the JAK-STAT pathway in VSMCs, triggering IL-8 release and MMP-9 activation to degrade matrix Gla protein (MGP), thereby facilitating hydroxyapatite deposition (39). Clinically, the phosphate binder sucroferric oxyhydroxide (SO) reduces serum IL-8 levels and attenuates calciprotein particle (CPP)-mediated endothelial inflammation in dialysis patients (46). Genetic interventions, such as GALNT3 overexpression, mitigate VC by O-GalNAc glycosylation of TNFR1 to inhibit NF-κB signaling and downregulate IL-8 expression (10). Furthermore, neutralization of IL-8 via monoclonal antibodies reverses the pro-calcific effects of uremic toxins like lanthionine, highlighting IL-8's role as a mediator of toxin-driven mineralization (47). These findings position IL-8-CXCR1/2 axis inhibition and upstream JAK-STAT/NF-κB modulation as promising strategies to disrupt inflammation-mediated calcification cascades.

#### IL-17

The IL-17 family cytokines, particularly IL-17A and IL-17F, promote VC by amplifying oxidative stress and inflammatory cascades. *In vitro* studies reveal that IL-17A enhances aortic calcification in a dose-dependent manner, primarily through inducing reactive oxygen species (ROS) and activating the Wnt/β-catenin pathway, which drives osteogenic differentiation of VSMCs (12). In chronic inflammatory skin disorders such as psoriasis and atopic dermatitis, overexpression of IL-17A/F exacerbates endothelial dysfunction and arterial stiffness, thereby accelerating atherosclerosis (45). Animal studies demonstrate a dose-dependent pro-calcific effect of IL-17A in murine ex vivo aortic calcification models. Intriguingly, another study reported that IL-17A requires co-application with IP-10 (CXCL10) to promote coronary artery calcification *in vitro*, suggesting a potential role of coordinated regulation by endothelial or inflammatory cells (48). Neutralizing IL-17A reduces neutrophil infiltration and aortic oxidative stress, restoring vascular elasticity (49). Clinically, IL-17 inhibitors (e.g., secukinumab) ameliorate both psoriatic lesions and atherosclerotic plaque burden, highlighting their potential cardioprotective effects (50). Collectively, targeting IL-17 receptors and downstream JAK/STAT signaling represents a promising therapeutic strategy to mitigate calcification associated with chronic inflammatory and metabolic disorders.

#### IL-24

IL-24, a pro-inflammatory cytokine, has recently emerged as a potent driver of VC through multi-pathway activation. Kawada et al. first demonstrated that iron overload synergizes with TNF-α to upregulate IL-24 expression in human aortic smooth muscle cells (HASMCs), inducing calcification that is reversible by anti-IL-24 antibodies, establishing IL-24 as a critical mediator of iron-dependent mineralization. Mechanistically, IL-24 activates the STAT3 signaling pathway, upregulating osteogenic markers Runx2 and BMP-2, while enhancing the Wnt/β-catenin axis to promote osteochondrogenic transdifferentiation of VSMCs (51). On this basis, studies have shown that the expression of osteogenic markers is increased in the aortic tissue of iron overload rats, and the increase of IL-24 may play a role in the process of iron promoting calcification (52). Clinically, IL-24 is overexpressed in calcified vessels of CKD patients, correlating positively with serum iron levels and inflammatory markers (e.g., hsCRP), suggesting its potential as a biomarker for iron dysregulation-associated calcification. However, the spatiotemporal specificity of IL-24 receptor signaling in atherosclerotic calcification remains poorly defined, and targeted therapies face challenges such as receptor promiscuity and inefficient nanoparticle delivery across calcified plaques.

IL-24 engages in bidirectional crosstalk with classical inflammatory and metabolic pathways through context-dependent mechanisms. In Th17 cells, IL-17A induces IL-24 via NF-κB activation, creating an autocrine negative feedback loop that suppresses GM-CSF and IL-17F production to limit immunopathology (53). Paradoxically, IL-24 itself promotes mitochondrial STAT3 accumulation through interaction with Grim19 (a complex I component), driving IL-10 production that further constrains Th17 pathogenicity (54). In stromal compartments, IL-17A directly upregulates IL-24 in skin fibroblasts and keratinocytes, amplifying keratinocyte proliferation in psoriasis through coordinated induction of IL-19/IL-24 (55). Hypoxia integrates with this signaling via HIF-1α stabilization, which converges with STAT3 activation in epithelial progenitors to induce IL-24 during tissue repair (56). Pathologically, IL-24 exacerbates renal fibrosis by inducing TGF-β1, PDGF-B, and CTGF in tubular cells, while IL-20 receptor beta (IL-20RB) deficiency attenuates fibrotic gene expression in obstructive nephropathy (57). This positions IL-24 as a nodal regulator bridging immune activation, metabolic stress, and fibrocalcific responses.

#### IL-29

IL-29, a member of the type III interferon family, has recently emerged as a key driver of VC through activation of the JAK2/STAT3/BMP2 signaling axis. Previous studies have shown that IL-29 inhibits osteoclastogenesis by activating the STAT signaling pathway, blocking NF-κB activation and NFATc1 translocation, and inhibiting downstream osteoclastogenic gene expression (58). According to a recent study reveals elevated IL-29 expression in calcified carotid arteries of patients with coronary artery disease or CKD, where it positively correlates with bone morphogenetic protein 2 (BMP2) level (23). Mechanistically, IL-29 binds to its specific receptor IL-28Rα, triggering JAK2/STAT3 pathway activation, which induces osteogenic transdifferentiation of VSMCs and accelerates hydroxyapatite deposition. *in vitro* and ex vivo studies demonstrate that pharmacological inhibition of IL-28Rα or JAK2 significantly attenuates VSMC calcification and suppresses BMP2 expression, highlighting the therapeutic potential of targeting the IL-29/IL-28Rα axis to disrupt calcification cascades in vascular pathologies.

IL-29 mediates bidirectional regulation through crosstalk with canonical inflammatory pathways. In the osteoarthritis microenvironment, IL-29 significantly enhances synovial fibroblast production of IL-1β, IL-6, and MMP-3 through MAPK/NF-κB pathways (but not JAK-STAT), accelerating cartilage degradation (59). This tissue-specific regulatory pattern demonstrates IL-29's capacity to selectively engage STAT or MAPK/NF-κB pathways according to microenvironmental context, enabling precise immunomodulation balancing immune homeostasis and inflammatory responses.

### Anti-calcification mechanisms and therapeutic targeting in vascular pathology

#### IL-10

IL-10, a pivotal anti-inflammatory cytokine, exerts inhibitory effects on VC by suppressing pro-inflammatory signaling and modulating mineralization homeostasis. In Apolipoprotein E knockout (ApoE^−/−^) mice, exogenous inorganic pyrophosphate (PPi) supplementation significantly elevates serum IL-10 levels while reducing pro-calcific cytokines such as TNF-α and IL-6, thereby attenuating atheromatous calcification progression (60). Mechanistically, IL-10 activates the STAT3/SOCS3 axis to inhibit NF-κB-driven transcription, downregulating osteogenic differentiation markers (e.g., Runx2, BMP2) in VSMCs (61). Preclinical studies further demonstrate that T cell-mediated immunomodulation (e.g., mCRAMP immunization) enhances IL-10 secretion by CD8^+^ T cells, reducing atherosclerotic plaque calcification incidence from 56% to 0% (*p* = 0.003) and fostering an anti-inflammatory microenvironment (62). Additionally, IL-10 upregulates Klotho expression, counteracting FGF23 signaling to preserve calcium-phosphate equilibrium and inhibit hydroxyapatite crystallization. These findings underscore the therapeutic potential of IL-10-targeted strategies—including recombinant IL-10 administration or PPi mimetics—to disrupt both inflammatory and mineralization cascades in VC.

### Dual roles in vascular calcification: context-dependent mechanisms and therapeutic implications

#### IL-4 and Il-13

IL-4 and IL-13, as Th2 cytokines, exhibit spatiotemporal dual roles in VC through microenvironment-dependent mechanisms. A 2023 study demonstrated that eosinophil-derived cationic proteins (e.g., ECP) directly promote vascular smooth muscle cell (VSMC) osteogenic differentiation via the BMPR-1A/1B-Smad1/5/8-Runx2 axis, while IL-4 and IL-13 showed no direct pro-calcific effects in this process (63). Conversely, in diabetic models, IL-13 drives VSMC osteogenic transdifferentiation through the CHI3L1-IL-13Ra2-JAK1-STAT3 pathway, with H3K18 lactylation amplifying this signal to increase calcification (64). Paradoxically, IL-4 correlates with hand VC severity and all-cause mortality in rheumatoid arthritis (RA) patients (HR = 1.41, 95% CI 1.12, 1.78; *P* = 0.004) (65), yet short-term IL-4 exposure upregulates osteoprotegerin (OPG) via STAT6 to inhibit calcification, whereas chronic exposure induces Cbfa1-mediated osteogenic differentiation (66). Furthermore, alternative macrophages (M2) in calcified plaques express elevated IL-4 receptors and suppress osteoclastic activity via IL-10 secretion (67), while IL-13 promotes calcification through crosstalk between WNT3/β-catenin and STAT3 pathways (32). This functional dichotomy arises from metabolic heterogeneity: hyperlactate diabetic microenvironments epigenetically enhance IL-13 signaling, whereas chronic inflammation balances IL-4-driven fibrotic and anti-calcific responses via M2 polarization. Therapeutic strategies targeting IL-13Ra2 antagonism or H3K18 lactylation inhibition may offer precision interventions to mitigate VC progression.

#### IL-18

IL-18, a pro-inflammatory cytokine, exhibits context-dependent dual roles in VC, with its effects intricately linked to microenvironmental signaling crosstalk. Clinical studies demonstrate a strong positive correlation between serum IL-18 levels and coronary artery calcium scores (r = 0.91, *p* < 0.001), mediated via TRPM7 channel activation and ERK1/2 signaling, which upregulate osteogenic markers Runx2 and BMP-2 in VSMCs (68, 69). In CKD, IL-18 exacerbates aortic calcification through p38 MAPK pathway activation, correlating with increased aortic pulse wave velocity (aoPWV) (29, 70). Mechanistically, IL-18 enhances VSMC osteochondrogenic transdifferentiation via SGK1-dependent pathways, while SGK1 inhibition attenuates calcification (71). Furthermore, IL-18 amplifies pyroptosis by activating the NLRP3/caspase-1/GSDMD axis, fostering a pro-calcific microenvironment (11, 36). Paradoxically, IL-18-driven inflammatory responses may indirectly suppress calcification by inducing autophagic clearance of apoptotic bodies (72). Notably, Elabela counteracts IL-18-associated cuproptosis and VC by activating PPAR-γ/FDX1 signaling (37). These paradoxical effects underscore IL-18's spatiotemporal duality: while predominantly pro-calcific via TRPM7, SGK1, and pyroptotic pathways, its inflammatory milieu may trigger compensatory anti-calcific mechanisms. Targeting downstream effectors (e.g., TRPM7 inhibitors, SGK1 antagonists) rather than IL-18 itself may optimize therapeutic precision, balancing anti-inflammatory efficacy with calcification mitigation.

#### IL-37

IL-37, a unique anti-inflammatory member of the IL-1 cytokine family, emerging evidence highlights IL-37's dual role in VC, balancing anti-inflammatory protection with compensatory biomarker elevation in advanced disease. Preclinical studies demonstrate IL-37 suppresses pro-calcific pathways by inhibiting endothelial-to-mesenchymal transition (EndMT) through Notch1/p38 MAPK-NF-κB signaling, thereby reducing smooth muscle cell activation and extracellular matrix remodeling in coronary artery model (73). This aligns with findings that IL-37 attenuates ox-LDL-induced endothelial osteogenic differentiation and endoplasmic reticulum stress via Smad3 binding, suggesting direct anti-calcification mechanisms (74). However, clinical observations reveal elevated plasma IL-37 levels in patients with severe coronary artery calcification (CAC), showing positive correlations with Agatston scores and inflammatory markers like hsCRP (75, 76). This paradox may reflect IL-37's compensatory upregulation in chronic inflammation, as evidenced by its predominant expression in macrophages and VSMCs within calcified lesions. While IL-37 demonstrates therapeutic potential in mitigating early calcification drivers like EndM (73), its sustained elevation in advanced disease might indicate either a failed protective response or biomarker utility for monitoring calcification progression (13). These findings underscore the need for phase-specific evaluation of IL-37's role in VC pathophysiology.

### Roles that may be overlooked

#### IL-33

IL-33, a pro-inflammatory cytokine of the IL-1 family, although direct evidence linking IL-33 to VC remains limited, emerging insights into its receptor ST2 suggest potential regulatory roles in calcification-related pathways. The IL-33/ST2 axis has been implicated in fibrocalcific processes, as soluble ST2 (sST2) correlates with coronary artery calcium scores and serves as a biomarker for atherosclerosis severity (77). In carotid atherosclerotic plaques, transmembrane ST2l overexpression on macrophages in symptomatic patients suggests its involvement in plaque destabilization, potentially creating a microenvironment conducive to calcification (78). Notably, IL-33 binding to ST2l exhibits cardioprotective effects by counteracting fibrosis in rheumatic valvular disease, while sST2 acts as a decoy receptor that exacerbates fibrotic remodeling—a precursor to calcification (79). This dual receptor dynamic may influence VC progression, particularly through macrophage-mediated inflammatory pathways. In peripheral arterial disease, IL-33 co-localizes with NLRP3 inflammasome components in calcified vessels, implicating IL-33-associated inflammation in calcification initiation (80). However, the therapeutic potential of modulating this axis remains unclear, as ACE inhibitors reduce sST2 levels but show limited correlation with calcification regression in clinical trials (81). These findings collectively suggest IL-33 may exert context-dependent effects on VC through receptor-specific signaling and inflammatory crosstalk, warranting further mechanistic investigation.

**Figure 2:**
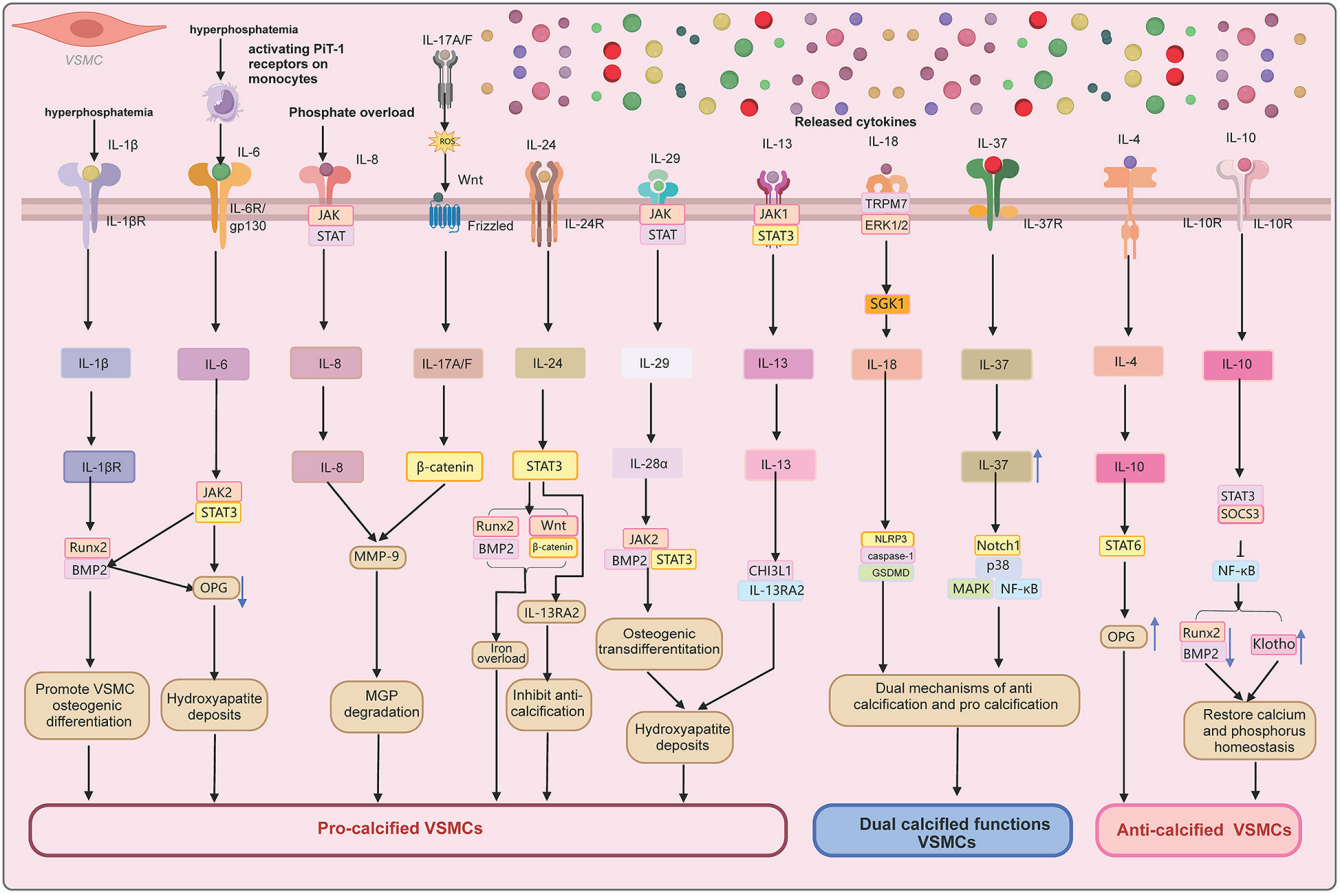
Molecular pathways of IL cytokines in VC.

This Figure 2 illustrates the interplay between inflammatory cytokines and osteogenic/chondrogenic transdifferentiation in VC. Pro-inflammatory cytokines (e.g., IL-1, IL-6, TNF-α) activate NF-κB and MAPK pathways, driving VSMCs toward an osteoblast-like phenotype and promoting calcium phosphate deposition. Key regulatory factors (e.g., OPG/RANKL, MGP) modulate these processes, either facilitating or inhibiting calcification. Exogenous stimuli (e.g., oxidative stress, dyslipidemia) exacerbate this cycle, leading to vascular dysfunction and disease progression. This model highlights the inflammatory-calcification crosstalk and potential therapeutic targets.

This Table 1 briefly outlines the pro-calcific or anti-calcific roles of various ILs in the VC process, along with their principal molecular pathways (e.g., BMP2/Runx2, JAK/STAT, Wnt/β-catenin, and the NLRP3 inflammasome) and clinical relevance. Reference numbers are provided for further consultation.

**Table 1:** Role of IL family in the process of VC.

IL Family member	Role in VC	Mechanistic pathways	Clinical relevance	Cited authors
IL-1β	Pro-calcific	Activates NLRP3 inflammasome-pyroptosis (gasdermin D); upregulates Runx2/BMP2 via NF-κB; TCF21 feedforward loop	Elevated in CKD patients; NLRP3 inhibition (MCC950) and ligustrazine reduce calcification	(9, 11, 34–37)
IL-6	Pro-calcific	JAK2/STAT3 activation; TCF21-ERK1/2-β-catenin synergy; suppresses OPG	High IL-6 tertiles predict 2.2-fold mortality risk in CKD; JAK inhibitors (tofacitinib) show efficacy	(9, 22, 25, 38–44)
IL-8	Pro-calcific	JAK-STAT-mediated MMP-9 activation; GALNT3 overexpression inhibits NF-κB	Phosphate binders (sucroferric oxyhydroxide) reduce IL-8 in dialysis patients	(10, 39, 45–47)
IL-17A/F	Pro-calcific	Wnt/β-catenin & ROS pathways; synergizes with IP-10 (CXCL10) for coronary calcification	IL-17 inhibitors (secukinumab) reduce plaque burden in psoriasis	(12, 45, 48–50)
IL-24	Pro-calcific	STAT3/Wnt activation; iron overload synergizes with TNF-α	Overexpressed in CKD calcified vessels; correlates with serum iron/hsCRP	(51–57)
IL-29	Pro-calcific	JAK2/STAT3/BMP2 axis via IL-28Rα receptor	Elevated in CAD/CKD carotid arteries	(23, 58, 59)
IL-10	Anti-calcific	STAT3/SOCS3-mediated NF-κB suppression; Klotho upregulation	Exogenous pyrophosphate elevates IL-10, reducing calcification in ApoE^−/−^ mice	(60–62)
IL-4/IL-13	Dual role	IL-13: CHI3L1-IL-13Ra2-JAK1-STAT3; IL-4: STAT6-OPG transient inhibition	IL-4 correlates with hand VC severity in RA (HR = 1.41 mortality)	(63–67)
IL-18	Dual role	TRPM7/ERK1/2 & SGK1 pathways; NLRP3/caspase-1/GSDMD pyroptosis	Serum IL-18 correlates with coronary calcium scores (r = 0.91); Elabela counteracts	(11, 29, 36, 37, 68–72)
IL-37	Dual role	Inhibits EndMT via Notch1/p38 MAPK-NF-κB; Smad3 binding in ox-LDL stress	Elevated in severe CAC patients; paradoxically linked to hsCRP	(13, 73–76)
IL-33	Indirect pro-calcific	sST2 (decoy receptor) correlates with atherosclerosis; co-localizes with NLRP3	IL-33/ST2l axis implicated in plaque destabilization	(77–81)

HR, indicates hazard ratio; and RR, relative risk.

### Prospects and challenges

The IL family exhibits marked functional heterogeneity in VC, dynamically regulated by microenvironmental cues. Functional heterogeneity within vascular microenvironments critically regulates calcification processes through dynamic interactions between cellular components and extracellular matrix (ECM) signaling. Single-cell proteomic profiling reveals that smooth muscle cell (SMC) phenotypic switching under altered shear stress involves Notch1/p38 MAPK-NF-κB signaling, with IL-37 suppressing pro-inflammatory EndMT to mitigate calcification (73). The ECM composition directly modulates SMC behavior, as demonstrated by reduced LTBP1 expression in unstable atherosclerotic plaques, which promotes SMC calcification through disrupted TGF-β signaling (82). NLRP3 inflammasome activation in macrophages adjacent to calcified areas creates a pro-osteogenic niche via IL-1β and TNF-α secretion, with pharmacological inhibition of NLRP3 attenuating VC in CKD models (35, 80). Microenvironmental phosphate overload induces SMC pyroptosis through potassium efflux-dependent NLRP3 activation, independent of canonical IL-1β signaling (83). Dynamic co-culture models reveal endothelial-SMC crosstalk amplifies calcification through TGF-β1/SIRT1 axis modulation, highlighting microenvironment-dependent phenotypic plasticity (84). Exosomal miR-302d-5p from endothelial cells suppresses SMC osteogenesis via Wnt3 inhibition in a m6A-dependent manner, illustrating epigenetic regulation within the vascular wall microenvironment (85). These findings underscore the spatial and temporal complexity of microenvironmental regulation in VC.

### Precision therapeutic strategies

#### Innovations in nanodelivery systems

Recent advancements in nanodelivery systems have significantly enhanced the targeting efficacy and therapeutic outcomes for VC. For calcific aortic valve disease (CAVD), Chen et al. developed PAR2-targeted magnetic nanocargoes that achieved dual-active targeting through PAR2-specific hexapeptides and magnetic field navigation, effectively suppressing osteogenic differentiation of valvular interstitial cells and alleviating calcification in *Ldlr(-/-)*mice (86)^.^ In abdominal aortic aneurysm (AAA) therapy, Hu et al. engineered α-cyclodextrin-based LaCD nanoparticles, which mitigated neutrophilic inflammation and NETosis, significantly improving vascular structural integrity. Functionalization with alendronate further enhanced targeting capability and therapeutic efficacy (87). For diabetic VC, Li et al. designed mitochondria-targeted nanodrugs (T4O@TPP/PEG-PLGA) that utilized TPP ligands for precise mitochondrial delivery, reducing oxidative stress induced by hyperglycemia and restoring mitochondrial morphology in animal models (88). Additionally, Mo et al. reported a dual-targeting virus-like nanocage (EVMS@R-HNC) that bound integrin αvβ3 on macrophages and smooth muscle cells, synergistically inhibiting inflammatory microenvironment dysregulation while preserving contractile function, offering a multifaceted solution for complex vascular pathologies (89). Zhang et al. further demonstrated through spatial conformation analysis that cyclic RGD exhibited superior αvβ3 integrin binding specificity compared to linear RGD, providing molecular insights for optimizing targeted nanocarrier design (90)^.^

But nanocarriers face significant challenges in targeting efficiency and biosafety. PEGylated and CD-47-functionalized magnetic nanoporous silica nanoparticles paradoxically accumulated primarily in the liver and spleen (86% of administered dose) rather than infected implant sites, with severe inflammation-driven off-target distribution in murine models (91). Polymeric PLGA-PEG nanoparticles induced sublethal hepatotoxicity *in vitro*, where redox-responsive variants (RR-NPs) triggered reactive oxygen species (ROS) surges and impaired albumin/urea production, while non-redox-responsive nanoparticles (nRR-NPs) additionally caused DNA damage in hepatocytes despite comparable cellular uptake (92). These findings underscore unresolved targeting inaccuracies and organ-specific toxicity risks.

#### Potential for multi-target synergistic intervention

Multi-target synergistic strategies demonstrate promising potential in addressing the multifactorial pathogenesis of VC. Aierken et al. identified that inhibition of iron transporters Slc39a14/Slc39a8 alleviated ferroptosis in VSMCs by mitigating iron overload, revealing novel targets at the intersection of metabolism and oxidative stress (93). Chen et al. demonstrated that oleoylethanolamide (OEA) suppressed STING1-mediated mitochondrial DNA damage and cellular senescence via Nrf2 activation while modulating the PPARα-dependent autophagy-ferroptosis axis, achieving multi-dimensional anti-calcification effects in diabetic models (94). Shen et al. reported that the natural compound thonningianin A activated L-type calcium channels to induce autophagy, downregulating RUNX2/BMP2 expression, and validated its synergistic effects via Cav1.2 α1C targeting in type 2 diabetes mellitus (T2DM) models (95). Furthermore, Sun et al. discovered that TFEB activation enhanced autophagic flux and the Nrf2 antioxidant system, suppressing diabetic VC through the POSTN-TGFβR1-YAP/TAZ pathway, highlighting the integration of transcriptional regulatory networks for multi-target intervention (96). These studies collectively suggest that coordinated modulation of metabolic dysregulation, oxidative stress, and epigenetic remodeling may form the cornerstone of future precision therapies.

#### Clinical challenges in balancing anti-inflammatory and immunosuppressive risks

The central role of anti-inflammatory therapy in managing chronic inflammatory diseases necessitates a delicate balance with immunosuppression-related risks. Datta-Mannan et al. reported that the oral IL-17A small-molecule inhibitor LY3509754 significantly reduced IL-17A activity but induced lymphocytic hepatitis and drug-induced liver injury (DILI) in high-dose cohorts (400–1,000 mg), underscoring the imperative for rigorous hepatic safety monitoring when targeting the IL-17 pathway (97). In phase III trials for hidradenitis suppurativa, Kimball et al. demonstrated that the dual IL-17A/F inhibitor bimekizumab achieved a 52% HiSCR50 response rate, yet elevated risks of oral candidiasis and opportunistic infections highlighted the need for immune status-based dosing optimization (98). A Nordic multicenter study by Glintborg et al. comparing secukinumab with TNF inhibitors revealed doubled hospitalization rates for infections with secukinumab (IR 5.0 vs. 2.3/100 patient-years for adalimumab), though attenuated after confounding adjustment, advocating personalized infection risk stratification (99). Azadeh et al. meta-analysis of IL-17 inhibitors in ankylosing spondylitis identified significantly increased mucosal/opportunistic infection risks (RD = 0.09, *p* = 0.02), recommending adjunct antifungal prophylaxis (100). Smolen et al. found the IL-6 inhibitor olokizumab non-inferior to adalimumab in rheumatoid arthritis but associated with higher infection rates (∼70%), emphasizing the need to reconcile target specificity with systemic immunosuppression (101). Collectively, precision biomarker stratification (e.g., IL-23/Th17 axis activity), dynamic immune monitoring, and stepwise dose titration emerge as critical strategies to optimize the anti-inflammatory-immunosuppression risk-benefit ratio.

## Conclusion

The roles of IL family members in VC are complex and diverse. They can either exacerbate the calcification process by promoting inflammation and cell transformation or protect against it by modulating immune responses and reducing oxidative stress. Given the crucial role of IL signaling pathways in calcification, targeting IL family signaling pathways holds significant therapeutic potential. Inhibiting pro-calcification cytokines or activating protective cytokine pathways could provide novel interventions for VC-related diseases. However, to achieve this goal, further in-depth research is needed to better understand the mechanisms of action, signaling networks, and relationships of IL family members with VC, to develop more effective targeted strategies for clinical treatment.
